# Exploring the association of mindfulness, confidence, competitive state anxiety, and attention control in soccer penalty shootouts

**DOI:** 10.3389/fpsyg.2024.1439654

**Published:** 2024-09-12

**Authors:** Lisi Shi, Longjun Jing, Huilin Wang, Yang Liu

**Affiliations:** ^1^School of Physical Education, Hunan University of Science and Technology, Xiangtan, China; ^2^School of Business, Hunan University of Science and Technology, Xiangtan, China; ^3^School of Social and Political Science, The University of Edinburgh, Edinburgh, United Kingdom

**Keywords:** soccer penalty shootout, mindfulness, confidence, competitive state anxiety, attention control

## Abstract

**Introduction:**

Penalty shootouts are a pivotal factor influencing outcomes in soccer matches. Soccer players face the challenge of overcoming physical fatigue and psychological pressure when taking penalty kicks. Instances of low confidence and competitive state anxiety during matches exacerbate the detrimental impact on attention control, particularly in non-target defined features, leading to suboptimal performance during penalty kicks.

**Methods:**

This cross-sectional survey investigates the relationship between mindfulness and attention control. Convenient sampling was employed to gather a sample of 266 soccer players from sports teams and training bases in Central and South China who had participated in city-level or higher-level competitions. A structural equation model, created using AMOS 26, was employed for hypotheses validation.

**Results:**

Findings reveal a positive correlation between mindfulness and confidence, and a negative correlation between mindfulness and competitive state anxiety. Additionally, confidence positively associates with attention control, while competitive state anxiety shows a negative relationship with attention control. Confidence and competitive state anxiety also function as mediators in the correlation between mindfulness and attention control. To elaborate, soccer penalty kickers with heightened mindfulness demonstrate increased confidence levels, reduced competitive state anxiety, and improved attention control.

**Discussion:**

Mindfulness training positively influences attention control during penalty kicks for soccer players. To boost players’ confidence, alleviate competitive state anxiety, and enhance their performance during penalty shootouts, it is recommended that governments increase investment in mindfulness training and talent development. Coaches should enhance their understanding of mindfulness training mechanisms, and athletes themselves should prioritize mindfulness training.

## 1 Introduction

Football is hailed as the world’s foremost sport, boasting immense influence and a vast number of fans. In terms of commercial value and revenue, football rightfully deserves the title of the world’ premier sport. In football matches, penalty shootouts are the most riveting and thrilling moments. Whether it’ a penalty awarded during regular time or one taken during the decisive phase of a penalty shootout, both can directly impact the final result of the game and even determine the winner (Tuğlu et al., 2022).

Players shoot from the penalty spot, situated 11 meters (12 yards) from the midpoint between the two goalposts, aiming at a goal that is 7.32 meters (8 yards) wide and 2.44 meters (8 feet) high. Various observational studies, including video analysis of proficient penalty referees, qualitative studies interviewing expert referees, and laboratory-based experiments, indicate associations between certain behaviors and psychological variables and successful performance in football penalty shootouts (Wood et al., 2015). For example, Hill and Shaw (2013) conducted a study using a six-step approach (including crowd noise simulation, self-threat, coach evaluation, enforced targets, informing goalkeepers of the shot direction) to replicate real-world stressors and explore football players’ performance under high and low-pressure conditions. The study found that individual responsibility within the team (i.e., closed skills) often led to self-threat, making players perceive the most pressure (Hill and Shaw, 2013). As players walked away from midfield, the intensity of cognitive anxiety increased with perceived pressure, leading to more distractions (e.g., more thoughts about needing to score). When goalkeepers were informed of the shot direction for the last two penalty kicks, players perceived greater pressure, leading to a skill-focused attention, and their first shot performance was notably worse than their second consecutive shot, with the fourth shot showing a significant failure rate. Jordet et al. (2007) explored the impact of factors such as handling pressure, skill level, physical fatigue, and opportunities (e.g., goalkeeper movement direction) on football players’ performance in penalty shootouts during major international events from 1976 to 2004. The results suggested a negative correlation between the importance of the kick (handling pressure) and the outcome, while the correlation between skill level, fatigue, and the result was minimal or insignificant. In the study by Brinkschulte et al. (2023), an analysis of 1,711 penalty kicks taken during major international tournaments over 15 years revealed that high situational pressure increased the probability of completely missing a penalty by about 6%, regardless of the player’s skill level. Conversely, when a highly skilled player took the shot, the likelihood of the goalkeeper saving the penalty decreased by approximately 4%. The study concluded that high situational pressure reduces the probability of a successful penalty, while high skill level serves only as a buffer to mitigate the adverse effects of performance pressure. The extent to which these adverse factors, arising from the pressure of the game, affect football players depends largely on how they handle such situations (Ellis and Ward, 2022).

Penalties constitute a duel between the kicker and the goalkeeper, both attempting to anticipate each other’s intentions to secure victory (Wood and Wilson, 2010). Van Der Kamp (2006) conducted a live simulation experiment, examining the relative advantage of kickers adopting goalkeeper-independent and goalkeeper-dependent strategies during penalty kicks. Participants using the goalkeeper-independent strategy maintained a constant visual target, while those using the goalkeeper-dependent strategy predicted the goalkeeper’s save direction at different times before ball contact. The experiment results indicated that predicting goalkeeper actions increased the risk of missed shots, mainly due to continuous monitoring of goalkeeper actions, diverting attention from the ball, especially given the brief time available to adjust penalty kicks (Van Der Kamp, 2006). The phenomenon can be explained by spatial attention mechanisms (Shepherd et al., 1986). In other words, when observers search for specific targets in a visual scene, they typically direct attention toward items with known features, effectively concentrating attention (Bacon and Egeth, 1994). However, when ignored irrelevant stimuli capture attention, it can lead to impaired target detection (Folk et al., 2002). Especially in situations of match anxiety, penalty kickers may struggle to completely disregard the goalkeeper’s presence, focusing more on threatening stimuli posed by the goalkeeper, thereby compromising the original attention control system (Wilson et al., 2009). Therefore, penalty kickers need to establish attention control settings representing known attributes of the target to effectively guide attention to items with target-defining features.

Psychological traits (e.g., strong confidence, focused attention) and psychological skills (e.g., maintaining confidence, re-focusing attention) are crucial factors for promoting athletes’ optimal performance (Holland et al., 2010). Research indicates that mindfulness positively affects maintaining attention (Baltar and Filgueiras, 2018), enhancing attention control, reducing the risk of injuries in football players (Naderi et al., 2020), and lowering sports anxiety levels (Zadkhosh et al., 2018). Currently, mindfulness practices serve as a training method to improve athletes’ psychological skills (Birrer et al., 2012). For example, Mindfulness-Acceptance-Commitment (MAC) interventions, rooted in acceptance and commitment therapy, indirectly enhance athletes’ self-evaluation in sports training by refining mindfulness and emotion regulation, leading to improved sports performance (Josefsson et al., 2019). Norouzi et al. (2020) implemented an 8-week mindfulness-based stress reduction intervention for outstanding Iranian football players, finding that mindfulness training increased psychological flexibility in guiding attention, leading to increased efficiency in coping strategies and behavioral response flexibility for high-skill demand situations. Thus, in penalty situations, mindfulness is precisely the cognitive process football players need to cultivate.

Reviewing past research reveals that obstacles to football players’ penalty success mainly focus on the impact of skill tactics and other strategies, such as kicking motion (Lopes et al., 2014), ball contact area (Ishii et al., 2012), and penalty strategy (Noël et al., 2015). However, there is relatively less research on how psychological factors affect football players’ penalty performance. As a result, this study sets forth the following research objectives: (1) examine the correlation between mindfulness and football players’ confidence and competitive state anxiety during penalty kicks; (2) investigate the relationships between mindfulness and attention control in football players; (3) explore whether and how confidence, competitive state anxiety, and attention control interact; (4) provide recommendations for addressing psychological issues in football players during penalty kicks.

This study focuses on football players, considering the influence of attention control on the psychological pressure of penalty kicks and proposing mindfulness as an intervention to alleviate attention control. The specific pathway of analysis is as follows: mindfulness training (e.g., mindfulness-acceptance-commitment, mindfulness-based stress reduction, mindfulness meditation) is conducive to promoting football players’ optimal performance, thereby enhancing their self-acceptance. Confidence contributes to the emotional intelligence development of football players and reduces competitive state anxiety, thereby improving attention control during penalty kicks.

## 2 Literature review and hypotheses development

### 2.1 Mindfulness, confidence, attention control

Mindfulness is defined as the intentional maintenance of attention in the present task, non-judgmentally monitoring internal stimuli and external stimuli (Baer, 2003). Mindfulness features are described as observing or having a clear awareness of each presented experience (Creswell, 2017). Mindfulness interventions within clinical contexts predominantly involve mindfulness-based interventions and therapies (Orzech et al., 2009). Mindfulness training in Western healthcare is utilized to treat psychological disorders like depression and anxiety (Aherne et al., 2011). Similarly, mindfulness reduces perceived pressure and trait anxiety in choking athletes (Tang et al., 2023).

Confidence is a psychological structure that influences whether athletes succeed or not in sports (Short and Short, 2005). Specifically, it is a personality trait, reflecting trust in one’s ability to achieve a certain goal (Shrauger and Schohn, 1995). Lack of confidence leads to physiological reactions under the emotion of fear of performing poorly and failing, often diminishing the likelihood of success, especially in crucial performances, such as lawyers presenting cases to the Supreme Court, professional golfers approaching a game-winning shot, or penalty kickers (Compte and Postlewaite, 2004). Anari and Shafiei (2016) conducted a study with students from Azad University in Kerman city. The findings revealed a notable disparity in confidence scores between participants before and after the test, underscoring the pivotal role of mindfulness in augmenting confidence. Walker (2019) measured character mindfulness, psychological resilience-related confidence, and negative self-evaluation in provincial adolescent female field hockey players. Analyzing the correlation coefficients among variables, Walker concluded that character mindfulness was positively correlated with psychological resilience confidence in adolescent athletes, with self-evaluation as the mediator. Oguntuase and Sun (2022) identified self-control as an intermediary variable between mindfulness and confidence by conducting an 8-week mindfulness-acceptance-commitment intervention on elite football players, with a control group. The results revealed that mindfulness had a significant direct and indirect correlation with enhancing football players’ confidence. Therefore, this study proposes Hypothesis 1:

*Hypothesis 1 (H1)*: *There is a positive correlation between mindfulness and confidence*.

Attention control is typically perceived as a comprehensive process where external stimuli either attract or distract attention or are suppressed. Hakim et al. (2021) offer instances from driving, such as pertinent external cues like flashing warning signs or extraneous external distractions like flashing colored billboards, accompanied by pertinent experiments. These exemplify how attention control mechanisms assist in discerning which new information integrates with our ongoing working memory task, allowing attention to stay concentrated on the initial task. Individuals with poor attention control are more susceptible to anxiety and emotional distraction, attentional bias toward threatening stimuli, or efficiency deficits, especially in cognitive and motor performance (Young and Ellmers, 2022).

On the other hand, individuals possessing confidence tend to exhibit heightened proficiency and effectiveness in deploying the cognitive resources indispensable for attaining success in the realm of sports. This is because confident individuals can control attention in the problem-solving process when facing obstacles, while less confident individuals are more likely to focus on perceived imperfections for self-diagnosis (Hays et al., 2009). Tomé-Lourido et al. (2019) found that when athletes realize their performance does not meet activity requirements, confidence levels decrease. Their experimental research demonstrated a positive relationship between confidence and attention. Therefore, athletes can refocus skills by strengthening the relationship between attention control and confidence, avoiding choking. Junli et al. (2021) conducted a questionnaire survey on Chinese university students and, through smart-PLS analysis, proved a positive correlation between self-confidence and attention control among Chinese athletes. Self-motivation plays a significant moderating role between confidence and attention control. Therefore, this study proposes Hypothesis 2:

*Hypothesis 2 (H2)*: *There is a positive correlation between confidence and attention control*.

### 2.2 Mindfulness, confidence, competitive state anxiety, attention control

Mindfulness training can shift attention from the subjective evaluation of negative emotions to the fluctuation of bodily sensations, reducing the intensity of negative emotions such as fear and anxiety, and promoting psychological wellbeing (Farb et al., 2010). Previous studies often posit that competitive state anxiety in sports arises primarily from the perception of situational importance and the pressure generated by the uncertainty of outcomes. Competitive state anxiety exhibits a multidimensional nature, encompassing somatic anxiety characterized by physiological responses like increased heart rate and sweating, cognitive anxiety involving aspects such as self-doubt and fear of failure, and confidence entailing elements like reassurance and a sense of security (Eys et al., 2003). Cognitive anxiety embodies the psychological dimension of anxiety, entailing worry, negative self-talk, and distressing visual imagery. Conversely, somatic anxiety encompasses the physiological or emotional aspect, triggering responses like an accelerated heart rate, breathlessness, cold, and sweaty hands, as well as muscle tension (Burton, 1988). The multidimensional anxiety theory predicts that athletes’ expectations of success remain stable over time, with cognitive anxiety and confidence expected to remain stable in the period before the competition. However, as the competition approaches, somatic anxiety is anticipated to rapidly increase (Krane, 1994). Previous research suggests that mindfulness has anti-depressive and anti-anxiety effects. Interventions are effective in significantly alleviating overall psychological distress, particularly symptoms related to anxiety (Marchand, 2012).

Moreover, Tang et al. (2022), focusing on recovering athletes, found that mindfulness can reduce anxiety and fatigue when facing pressure, showing a significant negative correlation between them, particularly in athletes recovering from injuries. The analysis revealed a negative correlation between mindfulness and competitive state anxiety. Additionally, the implementation of mindfulness interventions holds promise in reducing competitive state anxiety among athletes (Li et al., 2023). Quantitative experiments on karate athletes demonstrated that Mindfulness-based sport performance enhancement training reduced competitive state anxiety, with anxiety reduction accompanied by increased confidence (Harita et al., 2022). Therefore, this study proposes Hypothesis 3:

*Hypothesis 3 (H3): There is a negative correlation between mindfulness and competitive state anxiety.*

Furthermore, athletes and coaches across various sports need to be attentive to the influence of anxiety and confidence levels on athlete performance (Habibi et al., 2017). Anxiety is typically categorized into trait anxiety, reflecting general anxiety features in personality, and state anxiety, representing temporary responses to specific situations (Endler and Kocovski, 2001). Competitive state anxiety is anxiety experienced by athletes in competitive situations, and it may be alleviated as athletes’ confidence levels increase. Past research has validated this, such as Finkenberg et al. (1992), who tested competitive state anxiety in cheerleading team members participating in the national university championships before the competition. The study delved into the intricate interplay between competitive state anxiety and the psychological as well as physiological dimensions of confidence, revealing a noteworthy negative correlation between competitive state anxiety and confidence. Chapman et al. (1997) concentrated on taekwondo athletes, employing a multivariate analysis of variance to assess Competitive State Anxiety Inventory-2 scores. The findings indicated that winners exhibited elevated confidence scores and lower competitive state anxiety scores compared to losers. In a recent investigation involving basketball players, Chun et al. (2022) probed the mediating impact of sports confidence on the association between competitive state anxiety and perceived performance. Their findings brought to light a negative correlation between confidence and competitive state anxiety. Consequently, Hypothesis 4 is postulated in the context of this investigation:

*Hypothesis 4 (H4): There is a negative correlation between confidence and competitive state anxiety.*

Attention control theory posits that anxiety reduces attention to the current task, increases attention to threat-related stimuli, and impairs attentional control (Eysenck et al., 2007). This theory is based on the assumptions of goal-directed attentional systems and stimulus-driven attentional systems (Coombes et al., 2009). Walker (2019) explored the impact of anxiety-induced attention changes on penalty kick performance, finding an increased influence of stimulus-driven attentional control systems during penalty kicks. Athletes under anxiety earlier or more intensely focused on salient stimuli, neglecting goal-driven and task-relevant stimuli, resulting in decreased attentional control and shooting performance. Presently, there is scarce research exploring the association between competitive state anxiety and attention control. Nevertheless, Tomé-Lourido et al. (2019), in their experimental investigation involving Spanish athletes, not only validated the previously mentioned positive correlation between confidence and attention control but also illustrated a negative correlation between competitive state anxiety and attentional control. Drawing from the attention control theory and the dynamics of penalty kicks, this study posits the Hypothesis 5.

*Hypothesis 5 (H5): There is a negative correlation between competitive state anxiety and attention control.*

### 2.3 The mediating roles of confidence and competitive state anxiety

**Figure 1:**
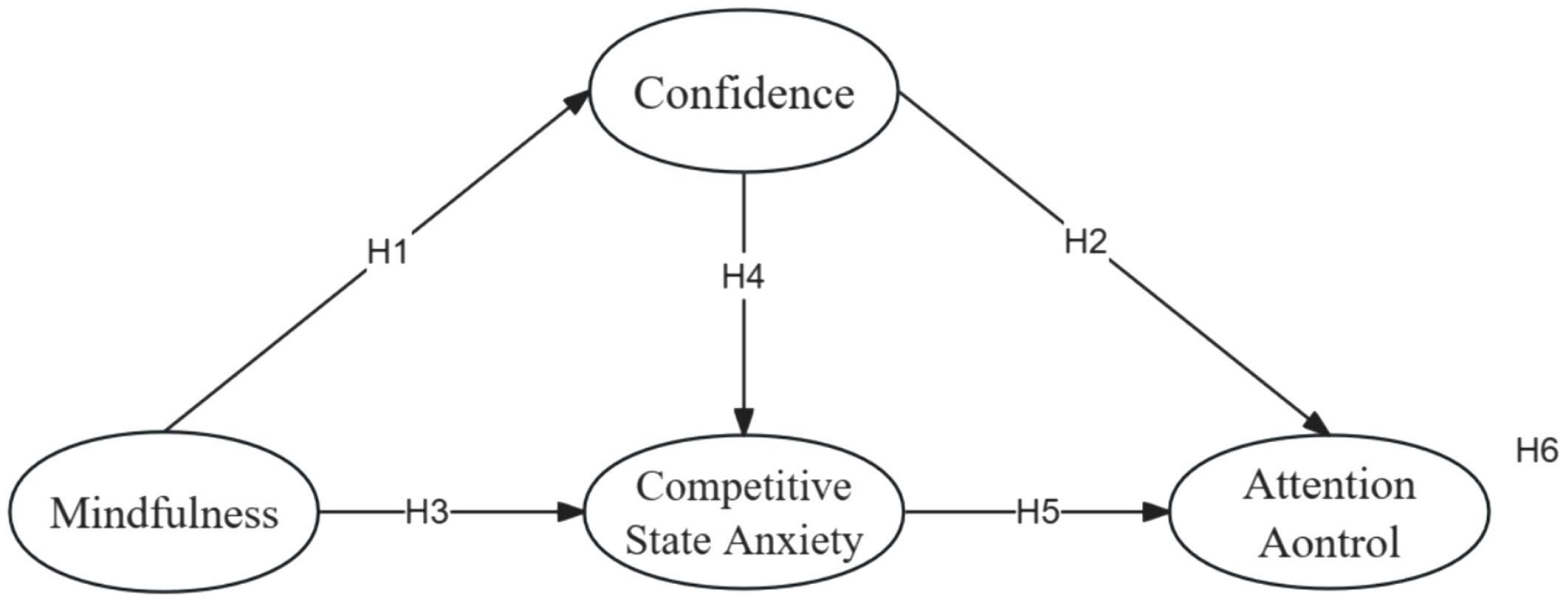
The conceptual framework.

Previous research indicates a close association between mindfulness and the improvement of attention control (MacDonald and Olsen, 2020). Mindfulness awareness is beneficial for maintaining sustained attention on a designated task and consciously shifting attention focus in the presence of distracting stimuli to maintain the current task (Chambers et al., 2008). Preliminary evidence suggests that mindfulness’s positive effects on emotional regulation and self-control can be realized by suppressing athletes’ negative emotions, reducing the cyclical nature of athlete anxiety (Friese et al., 2012). Mindfulness-acceptance-commitment intervention has been shown to effectively enhance athletes’ confidence (Oguntuase and Sun, 2022). Research indicates that athletes with lower confidence levels experience higher levels of competitive state anxiety (Harita et al., 2022), and lower levels of attention control (Junli et al., 2021). Individuals experiencing anxiety often exhibit heightened focus on processing threatening stimuli. This disruption leads to a decrease in attentional control, contributing to the phenomenon of choking (Clarke and Todd, 2021). Therefore, this study hypothesizes whether mindfulness can influence attention control through the mediating roles of confidence and competitive state anxiety (Figure 1). Based on this premise, the study proposes Hypothesis 6:

*Hypothesis 6 (H6): Confidence and competitive state anxiety mediate the relationship between mindfulness and attention control.*

## 3 Materials and methods

### 3.1 Procedure

This study targeted football players in the Central and Southern regions of China who had engaged in city-level or higher-level competitions and experienced penalty kicks or penalty shootouts during matches. It employed a combination of purposive and convenience sampling approaches. The researchers, during October to November 2023, briefed team leaders and coaches at various sports teams and training facilities in the Central and Southern regions about the purpose and nature of the study. They were requested to communicate the study’s objectives to the athletes and distribute the questionnaire among them once informed. Additionally, athletes were encouraged to share the questionnaire with their peers. Distributing a total of 300 questionnaires resulted in the collection of 266 valid responses, establishing an effective response rate of 88.7%.

Table 1 displays the demographic characteristics of the 266 athletes surveyed. Findings from the study indicate that: (1) nearly half of the surveyed individuals fall within the age range of 18–25 years. (2) The majority of participants in the study are male football players (55.4%), slightly outnumbering their female counterparts (44.6%). (3) More than half of the football players are university students. (4) In terms of skill level, close to half of the athletes (50.7%) are at the level of football players, with a small proportion (4.3%) reaching the level of elite athletes. (5) Over half of the football players have taken penalty kicks in game situations more than 10 times. (6) Approximately half of the football players participate in competitions at the provincial level (48.5%), with a smaller percentage (16.2%) competing at the national level.

**Table 1:** Characteristics of participants (*N* = 266).

Profiles	Survey (%)
*Age*	
18–25	151 (56.7%)
26–35	98 (36.8%)
≥36	17 (6.5%)
*Gender*	
Male	153 (57.6%)
Female	113 (42.4%)
*Education level*	
Below high school	17 (6.4%)
High school/vocational school	97 (36.3%)
College and above	152 (57.3%)
*Sport level*	
No sports grade certificate	119 (44.7%)
Second-level athlete	91 (34.2%)
Tier 1 athlete	46 (17.2%)
National athlete level	10 (3.9%)
*Number of penalties*	
1–5	51 (19.1%)
6–10	62 (23.3%)
11–15	68 (25.6%)
≥16	85 (32%)
*Event level*	
Municipal level	94 (35.3%)
provincial	129 (48.5%)
National level	43 (16.2%)

### 3.2 Measures

The survey employed in this study is composed of five sections, encompassing a total of 23 items. The initial segment of the questionnaire involved respondents furnishing demographic information such as age, gender, educational level, and sports proficiency. Subsequently, the second section gaged respondents’ mindfulness levels utilizing the five items from the Sport Mindfulness Scale (Thienot et al., 2014). Sample items include “I am able to notice the intensity of nervousness in my body.” In the third section, data on respondents’ self-esteem levels were gathered using four items from the Rosenberg Self-Esteem Scale (Heatherton and Wyland, 2003). An example item is “On the whole, I am satisfied with myself.” In the fourth section, participants’ competitive state anxiety was assessed using the revised Competitive State Anxiety-2 Scale (Martinent et al., 2010), which includes five items. Sample items include the statement “I feel self-confidence.” Lastly, the fifth section gathered data on respondents’ attention control using five items from the Attention Control Scale (Fajkowska and Derryberry, 2010). Sample items, such as “When trying to focus my attention on something, I have difficulty blocking out distracting thoughts.” were included. All four scales were assessed using a five-point Likert scale.

Pilot testing was then carried out to verify the reliability of the adapted survey instrument. Utilizing convenience sampling, 60 questionnaires were distributed to high-level university athletes in a specific city, resulting in 56 valid responses (Kimberlin and Winterstein, 2008). The outcomes revealed that Cα coefficients for all scales surpassed 0.9, denoting excellent internal consistency (Fornell and Larcker, 1981).

### 3.3 Data analysis

In this study, version 26.0 of AMOS was harnessed to construct a robust structural equation model (SEM) that delves into how mindfulness contributes to enhancing attention control among soccer penalty kick players. Adhering to the two-step modeling approach advocated by Anderson and Gerbing (1988), the evaluation encompassed both the measurement model and the structural model. Following a meticulous scrutiny of reliability and validity, the analysis proceeded to gage the fit and path coefficients of the hypothesized model while scrutinizing the presence of intermediate effects.

To address common method variation (CMV), this study implemented the recommended methodology, contrasting the chi-square values and degrees of freedom (df) between a single-factor model and a multifactor model. The results unveiled that the chi-square for the single-factor model was 1092.442 (df = 152, *p* < 0.001). In stark contrast, the chi-square for the multifactor model was 171.116 (df = 146, *p* < 0.001). This comparison indicates that the fit of the single-factor model aligns with that of the multifactor model. The outcomes strongly imply the absence of a single-factor structure, affirming that CMV exerts a negligible impact on this study and can be dismissed.

## 4 Results

### 4.1 Measurement model

In this study, the assessment of the reliability and discriminant validity of latent variables involved the calculation of Cα and CR coefficients. Table 2 presents the Cα coefficients, ranging from 0.834 to 0.901, with all CR values surpassing 0.8 for each variable. Moreover, the average variance extracted (AVE) for each variable fell within the range of 0.559–0.646. This indicates that all variables exhibited high reliability and convergent validity. Additionally, as per Table 3, all inter-variable correlation coefficients were below the square root of AVE, signifying outstanding discriminant validity among all variables.

**Table 2:** Reliability and validity.

Items	Loadings	Cα	AVE	CR
*Mindfulness (MIN)*		0.901	0.646	0.901
MIN1	0.818			
MIN2	0.818			
MIN3	0.825			
MIN4	0.783			
MIN5	0.774			
*Confident (CON)*		0.834	0.560	0.835
CON1	0.807			
CON2	0.716			
CON3	0.677			
CON4	0.787			
*Competitive state anxiety (CSA)*		0.868	0.559	0.863
CSA1	0.754			
CSA2	0.770			
CSA3	0.815			
CSA4	0.758			
CSA5	0.628			
*Attention control (AC)*		0.867	0.570	0.868
AC1	0.785			
AC2	0.724			
AC3	0.788			
AC4	0.787			
AC5	0.684			

**Table 3:** Evaluation of discriminant validity.

Construct	MIN	CON	CSA	AC
MIN	**(0.804)**			
CON	0.379 **	**(0.748)**		
CSA	−0.410 **	−0.533 **	**(0.748)**	
AC	0.429 **	0.551**	−0.508**	**(0.755)**

The diagonals of the matrix display the square root of the average variance extracted (bold), while off-diagonals represent Pearson’s correlation coefficients. **p < 0.01.

### 4.2 Hypothesis testing

The structural equation model demonstrated robustness as evidenced by high goodness-of-fit indices (*χ*^2^/df = 1.218, GFI = 0.934, AGFI = 0.915, NFI = 0.936, RMSEA = 0.029), surpassing recommended thresholds significantly. The Pearson correlation results in Table 3 revealed significant correlations among the independent, mediating, and dependent variables, thereby supporting the hypotheses.

**Figure 2:**
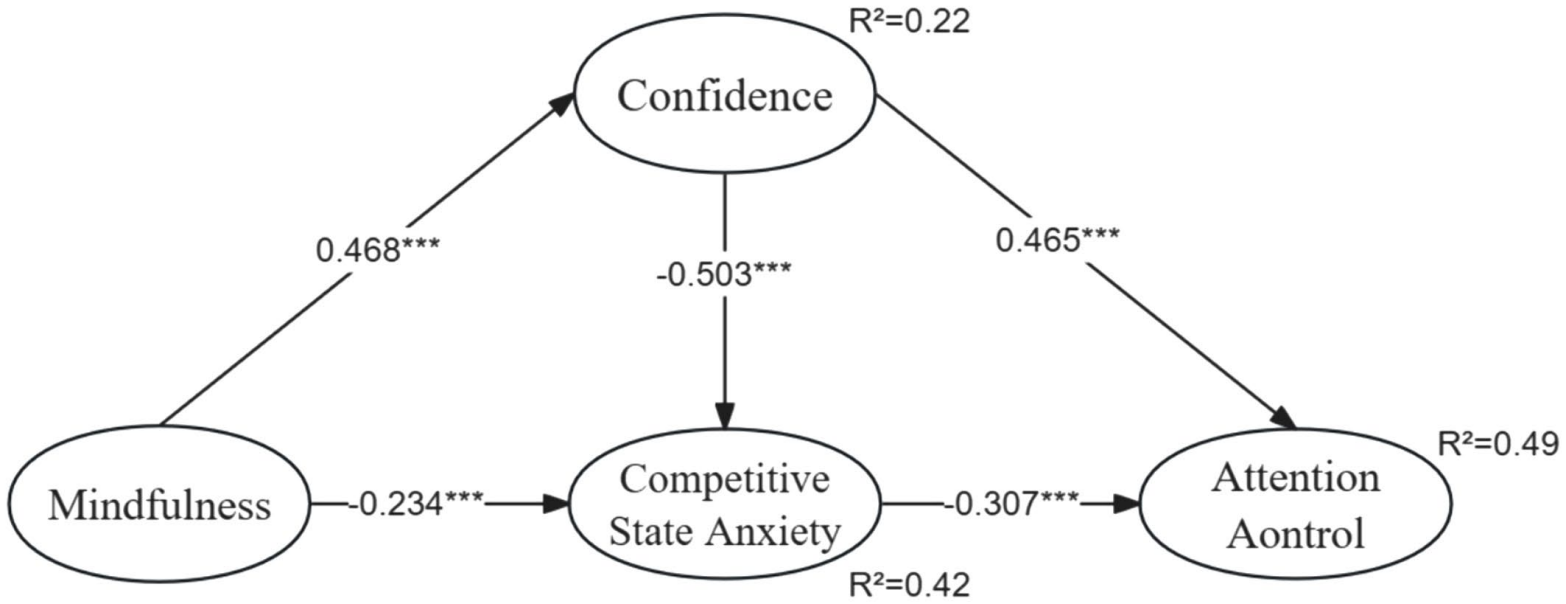
Structural model. ***p < 0.001.

The structural path model illustrated in Figure 2 revealed statistically significant relationships: the association between mindfulness and confidence was found to be significant (*β* = 0.468, *p* < 0.001), confirming H1; the relationship between mindfulness and competitive state anxiety was significant (*β* = −0.234, *p* < 0.001), supporting H2; the connection between confidence and competitive state anxiety was significant (*β* = −0.503, *p* < 0.001), substantiating H3; the association between confidence and attention control was significant (*β* = 0.465, *p* < 0.001), validating H4; and the link between competitive state anxiety and attention control was significant (*β* = −0.307, *p* < 0.001), affirming H5.

Hypothesizing that mindfulness influences soccer players’ attention control through the mediating pathways of confidence and competitive state anxiety, researchers employed Bootstrap resampling (5,000 samples) to scrutinize the mediation effects. The standardized results, along with a 95% confidence interval, are presented in Table 4. Notably, the *Z*-statistic absolute value for the MIN → AC mediation effect surpassed 1.96, indicating the exclusion of 0 within the 95% confidence interval.

**Table 4:** Indirect effects.

	Point estimate	Product of coefficients		Bootstrapping		
				Bias-corrected 95% CI		Two-tailed significance
		*SE*	*Z*	Lower	Upper	
MIN → AC	0.362	0.052	6.96	0.258	0.459	< 0.001

Furthermore, the association between mindfulness and attention control was significantly influenced by both confidence and competitive state anxiety (standardized indirect effect = 0.362, *p* < 0.001), corroborating H6. These results suggest that soccer players exhibiting higher levels of mindfulness, increased confidence, and decreased competitive state anxiety showcase improved attention control during penalty shootouts.

## 5 Discussion

### 5.1 Theoretical contribution

Firstly, existing research primarily focuses on the technical aspects (Lopes et al., 2014), target selection (Ishii et al., 2012), and strategies (Noël et al., 2015) during penalty kicks, with limited attention to the players’ attention control. This study addresses the issue of attention control among soccer players during penalty kicks, providing a more targeted perspective and enriching the relevant theoretical research. In soccer matches, scoring against the opponent within regular match time is challenging, involving intense physical confrontations and long-distance running. Whether it is a referee-awarded penalty or a penalty shootout, players have only a brief time to recover from physical and cognitive fatigue.

In high-pressure situations like a decisive penalty kick, players often struggle to adjust their confidence levels and manage competitive state anxiety when facing the goalkeeper. It is widely believed that selecting information from visual displays is controlled by both goal-directed and stimulus-driven mechanisms (Yantis, 1993). Anxious penalty takers are more influenced by the stimulus-driven attention control system, excessively focusing on the threatening goalkeeper, which can cause their shot to deviate from the intended target (Wilson et al., 2009). Additionally, studies have shown that confidence has a positive impact on sports performance, with a stronger relationship between confidence and performance in short-duration sports compared to long-duration sports (Lochbaum et al., 2022). This can be explained by spatial attention mechanisms: visual attention can be guided voluntarily or goal-directed by observers, or it can be stimulus-driven by attention capture (Serences et al., 2005).

Research indicates a positive correlation between confidence and attention control, and a negative correlation between competitive state anxiety and attention control, validating hypotheses H2 and H5. In other words, in high-pressure situations, it is more beneficial for penalty takers to maintain high confidence and low competitive state anxiety to increase their chances of success. Moreover, good psychological skills are crucial for successful penalty kicks (Junge et al., 2000). Considering the close relationship between threatening stimuli (e.g., negative emotions during the kick, the goalkeeper, noisy crowds) and optimal performance during penalties, as well as the tendency for distraction during the penalty moment to result in suboptimal performance, this study explores the necessity of interventions involving mindfulness and confidence to impact competitive state anxiety and attention control in football players.

The findings reveal a positive correlation between mindfulness and confidence and a negative correlation with competitive state anxiety (refer to Figure 2), aligning with the results observed by Oguntuase and Sun (2022) and Tang et al. (2022). Notably, mindfulness exerts the most significant influence on competitive state anxiety, followed by confidence. Thus, hypotheses H1 and H3 are supported. Penalty takers with mindfulness techniques are better equipped to quickly overcome confidence issues and anxiety, maintaining focused attention at the moment of the shot. Similarly, consistent with the studies by Finkenberg et al. (1992) and Chapman et al. (1997), this study found a negative correlation between confidence and competitive state anxiety, validating hypothesis H4. As the confidence level of penalty takers increases, symptoms of competitive state anxiety decrease.

Additionally, both confidence and competitive state anxiety act as mediators in the relationship between mindfulness and attention control. As illustrated in Figure 2, these variables collectively explain 49% of the variance in attention control, confirming hypothesis H6. The study thus offers a valuable pathway for exploring the connection between mindfulness and attention control, particularly by investigating the role of mindfulness intervention, starting from the lack of confidence and the occurrence of competitive state anxiety in penalty-taking soccer players.

### 5.2 Practical implications

The study highlights the significant correlation between mindfulness and the confidence and competitive state anxiety of soccer penalty kick-takers. It establishes a positive association between mindfulness and confidence and a negative association with competitive state anxiety. Furthermore, the research reveals how confidence and competitive state anxiety play a mediating role in the relationship between mindfulness and attention control. Given the myriad internal and external challenges faced by soccer penalty kick-takers during matches, coupled with the prevalence of unsuccessful penalties in soccer and their potential repercussions on athletes’ psychological wellbeing and performance, mindfulness emerges as a pivotal factor warranting consideration. In light of these insights, this study suggests that training facilities and coaches incorporate mindfulness training for soccer players to cultivate and enhance their mindfulness skills. Integrating mindfulness practices, such as meditation, into athletes’ training and pre-and post-match routines may improve confidence and effectively manage competitive state anxiety. These positive mindfulness interventions can also benefit athletes in other sports, particularly those susceptible to the influences of diminished confidence and anxiety, such as penalty takers or shooters. Thus, emphasizing the elevation of mindfulness training, especially for athletes displaying high levels of lack of confidence and anxiety, is essential.

However, the current status of mindfulness training for athletes in China is not ideal. This situation results from various factors, including coaches’ limited understanding of the principles and mechanisms of mindfulness training, inadequate training facilities, and a lack of professional mindfulness guidance courses. To address these challenges, the study proposes a series of feasible recommendations. To the government, it is recommended that the General Administration of Sport and relevant departments acknowledge the beneficial impacts of mindfulness on confidence, competitive state anxiety, and attention control. They should establish a professional system for training coaches in mindfulness, integrate relevant mindfulness training courses into university talent development programs, and increase the proportion of professionals proficient in mindfulness training. Simultaneously, greater financial investment in mindfulness training, designating some universities as mindfulness training and assessment bases, increasing the availability of related equipment, and establishing a mutually beneficial model between universities and soccer training facilities can be considered. Long-term monitoring and assessment of athletes’ psychological states by universities can enable coaches to develop more tailored training plans based on athletes’ specific conditions, further promoting the psychological health of soccer players.

Similarly, for coaches, enhancing their understanding of the principles and mechanisms of mindfulness training, as well as comprehending how mindfulness affects performance and reinforcing their grasp of mindfulness training methods, is crucial. Research has shown that mindfulness meditation practice is associated with improved attention control, with the shortest effective duration being 60 days. Additionally, the benefits of this practice tend to increase over time (Baltar and Filgueiras, 2018). Therefore, it is worth normalizing mindfulness meditation practice and integrating it into daily training routines. Furthermore, studies have demonstrated that the effectiveness of mindfulness interventions can be identified through monitoring cortisol levels and conducting continuous performance tests and attention capture tests (Meland et al., 2015). Coaches should prioritize athletes’ mental and physical health, promptly understanding their physical and mental states, analyzing them, and formulating corresponding improvement methods.

Recently, Vella-Fondacaro and Romano-Smith (2023) proposed a combined psychological skills training and mindfulness-based intervention program (PSTMI), with each session lasting 30 min. This program includes goal setting, imagery, arousal regulation, mindfulness practice, and self-talk, which are beneficial for better attention control and overall performance. In training and competition, coaches need to reasonably arrange the content of psychological skills training according to the training phase and competition level. For example, goal setting and imagery should be introduced early in pre-competition preparation to maintain athletes’ mental health, while mindfulness/arousal regulation and self-talk should be utilized during more challenging competitions to help athletes overcome the impact of threatening stimuli on their attention control.

According to the confirmed outcomes indicating that confidence and competitive state anxiety play a mediating role in the connection between mindfulness and attention control, athletes themselves should actively strive to deepen their comprehension of mindfulness principles. During mindfulness training, athletes should purposefully enhance their present-moment awareness, accept thoughts and emotions, and avoid reacting to stressors, to maintain focused attention during high-pressure penalty situations. In daily training, athletes should practice mindful meditation, focusing their thoughts on a single target, usually their breath, with firmness and clarity to achieve a calm mind and sustained concentration.

During competitions, especially before taking penalty kicks, athletes should utilize psychological skills such as mindfulness/arousal regulation and self-talk to overcome lack of confidence and competitive state anxiety when facing the goalkeeper, keeping their attention on the task at hand. Simultaneously, athletes should earnestly listen to and implement mindfulness techniques provided by coaches and psychological monitoring organizations to help overcome lack of confidence and competitive state anxiety, thereby improving attention control levels during penalty kicks.

## 6 Limitations

Firstly, the research model did not account for moderating variables such as athletes’ personalities and training levels. Future research should delve into potential variations and developments in the model. Secondly, the sample size only included soccer players from training bases and teams in the Central and South China regions. Consequently, the study results may not be extrapolated to more advanced soccer player cohorts or other sports. Subsequent research should encompass diverse levels of soccer populations, such as female soccer players or athletes from different sports. Lastly, due to the adoption of a cross-sectional design rather than an experimental longitudinal approach, the study’s breadth and depth are constrained. Future research could consider measuring pre-match, post-match, and in-match confidence and competitive state anxiety, dividing the samples into two groups, thereby enhancing the scientific rigor of the study.

## 7 Conclusion

Aligned with the research objectives, this study suggests that a significant portion of soccer players, when confronted with penalty kicks, can augment their attention control by modulating levels of confidence and competitive state anxiety through mindfulness training. The findings underscore the effectiveness of mindfulness training in improving soccer players’ attention control, especially under the intense pressure of penalty situations. Sustaining attention, boosting confidence, and alleviating competitive state anxiety are identified as crucial aspects where mindfulness training plays a significant role.

Mindfulness training enhances athletes’ confidence and alleviates competitive state anxiety, additionally influencing attention control levels through the mediating roles of confidence and competitive state anxiety. Therefore, monitoring soccer players’ ability to maintain attention under high-pressure environments through cortisol level monitoring, continuous performance tests, and attention capture tests is crucial. Implementing mindfulness meditation and mindfulness interventions can improve athletes’ psychological skills to handle the lack of confidence and competitive state anxiety triggered by penalty situations. On the field, athletes should consciously practice enhancing present-moment awareness, accepting thoughts and emotions, and not reacting to stressors to improve attention control levels. This practice helps maintain focused attention at the critical moment of taking a shot.

## Data Availability

The raw data supporting the conclusions of this article will be made available by the authors, without undue reservation.

## References

[ref1] Aherne C. Moran A. P. Lonsdale C. (2011). The effect of mindfulness training on athletes’ flow: an initial investigation. Sport Psychol. 25, 177–189. doi: 10.1123/tsp.25.2.177

[ref2] Anari A. M. Z. Shafiei Z. (2016). The effectiveness of mindfulness on motivation and academic achievement and increase confidence in students of Azad University of Kerman City, Iran, 2015. Eur. Psychiatry 33, S695–S696. doi: 10.1016/2Fj.eurpsy.2016.01.2070

[ref3] Anderson J. C. Gerbing D. W. (1988). Structural equation modeling in practice: a review and recommended two-step approach. Psychol. Bull. 103:411. doi: 10.1037/0033-2909.103.3.411

[ref4] Bacon W. F. Egeth H. E. (1994). Overriding stimulus-driven attentional capture. Percept. Psychophys. 55, 485–496. doi: 10.3758/BF03205306, PMID: 8008550

[ref5] Baer R. A. (2003). Mindfulness training as a clinical intervention: a conceptual and empirical review. Clin. Psychol. 10:125. doi: 10.1093/clipsy.bpg015

[ref6] Baltar Y. C. Filgueiras A. (2018). The effects of mindfulness meditation on attentional control during off-season among football players. SAGE Open 8:2158244018781896. doi: 10.1177/2158244018781896

[ref7] Birrer D. Röthlin P. Morgan G. (2012). Mindfulness to enhance athletic performance: theoretical considerations and possible impact mechanisms. Mindfulness 3, 235–246. doi: 10.1007/s12671-012-0109-2

[ref8] Brinkschulte M. Wunderlich F. Furley P. Memmert D. (2023). The obligation to succeed when it matters the most–the influence of skill and pressure on the success in football penalty kicks. Psychol. Sport Exerc. 65:102369. doi: 10.1016/j.psychsport.2022.102369, PMID: 37665841

[ref9] Burton D. (1988). Do anxious swimmers swim slower? Reexamining the elusive anxiety-performance relationship. J. Sport Exerc. Psychol. 10, 45–61. doi: 10.1123/jsep.10.1.45

[ref10] Chambers R. Lo B. C. Y. Allen N. B. (2008). The impact of intensive mindfulness training on attentional control, cognitive style, and affect. Cognit. Ther. Res. 32, 303–322. doi: 10.1007/s10608-007-9119-0, PMID: 27409075

[ref11] Chapman C. Lane A. M. Brierley J. H. Terry P. C. (1997). Anxiety, self-confidence and performance in tae kwon-do. Percept. Mot. Skills 85, 1275–1278. doi: 10.2466/pms.1997.85.3f.1275, PMID: 9450282

[ref12] Chun D.-R. Lee M.-Y. Kim S.-W. Cho E.-Y. Lee B.-H. J. I. J. O. E. R. Health P. (2022). The mediated effect of sports confidence on competitive state anxiety and perceived performance of basketball game. Int. J. Environ. Res. Public Health 20:334. doi: 10.3390/ijerph20010334, PMID: 36612655 PMC9819433

[ref13] Clarke P. J. Todd J. (2021). Lessons unlearned: a conceptual review and meta-analysis of the relationship between the attention control scale and objective attention control. Cogn. Emot. 35, 1447–1459. doi: 10.1080/02699931.2021.1987861, PMID: 34672869

[ref14] Compte O. Postlewaite A. (2004). Confidence-enhanced performance. Am. Econ. Rev. 94, 1536–1557. doi: 10.1257/0002828043052204, PMID: 38863152

[ref15] Coombes S. A. Higgins T. Gamble K. M. Cauraugh J. H. Janelle C. M. (2009). Attentional control theory: anxiety, emotion, and motor planning. J. Anxiety Disord. 23, 1072–1079. doi: 10.1016/j.janxdis.2009.07.009, PMID: 19674869 PMC2760607

[ref16] Creswell J. D. (2017). Mindfulness interventions. Annu. Rev. Psychol. 68, 491–516. doi: 10.1146/annurev-psych-042716-051139, PMID: 27687118

[ref17] Ellis L. Ward P. (2022). The effect of a high-pressure protocol on penalty shooting performance, psychological, and psychophysiological response in professional football: a mixed methods study. J. Sports Sci. 40, 3–15. doi: 10.1080/02640414.2021.1957344, PMID: 34847831

[ref18] Endler N. S. Kocovski N. L. (2001). State and trait anxiety revisited. J. Anxiety Disord. 15, 231–245. doi: 10.1016/S0887-6185(01)00060-3, PMID: 11442141

[ref19] Eys M. A. Hardy J. Carron A. V. Beauchamp M. R. (2003). The relationship between task cohesion and competitive state anxiety. J. Sport Exerc. Psychol. 25, 66–76. doi: 10.1123/jsep.25.1.66, PMID: 38974100

[ref20] Eysenck M. W. Derakshan N. Santos R. Calvo M. G. (2007). Anxiety and cognitive performance: attentional control theory. Emotion 7:336. doi: 10.1037/1528-3542.7.2.336, PMID: 17516812

[ref21] Fajkowska M. Derryberry D. (2010). Psychometric properties of attentional control scale: the preliminary study on a polish sample. Polish Psychol. Bull. 41, 1–7. doi: 10.2478/s10059-010-0001-7

[ref22] Farb N. A. Anderson A. K. Mayberg H. Bean J. Mckeon D. Segal Z. V. (2010). Minding one’s emotions: mindfulness training alters the neural expression of sadness. Emotion 10:25. doi: 10.1037/a0017151, PMID: 20141299 PMC5017873

[ref23] Finkenberg M. E. Dinucci J. N. Mccune E. D. Mccune S. L. (1992). Cognitive and somatic state anxiety and self-confidence in cheerleading competition. Percept. Mot. Skills 75, 835–839. doi: 10.2466/pms.1992.75.3.835, PMID: 1454483

[ref24] Folk C. L. Leber A. B. Egeth H. E. (2002). Made you blink! Contingent attentional capture produces a spatial blink. Percept. Psychophys. 64, 741–753. doi: 10.3758/BF03194741, PMID: 12201333

[ref25] Fornell C. Larcker D. F. (1981). Evaluating structural equation models with unobservable variables and measurement error. J. Mark. Res. 18, 39–50. doi: 10.1177/002224378101800104, PMID: 33691717

[ref26] Friese M. Messner C. Schaffner Y. (2012). Mindfulness meditation counteracts self-control depletion. Conscious. Cogn. 21, 1016–1022. doi: 10.1016/j.concog.2012.01.008, PMID: 22309814

[ref27] Habibi H. Moghaddam A. Soltani H. (2017). Confidence, cognitive and somatic anxiety among elite and non-elite futsal players and its relationship with situational factors. Pedagog. psychol. Med. Biol. Probl. Phys. Train. Sports 2, 60–64.

[ref28] Hakim N. Feldmann-Wüstefeld T. Awh E. Vogel E. K. (2021). Controlling the flow of distracting information in working memory. Cereb. Cortex 31, 3323–3337. doi: 10.1093/cercor/bhab013, PMID: 33675357 PMC8196257

[ref29] Harita A. N. W. Suryanto S. Ardi R. (2022). Effect of mindfulness sport performance enhancement (Mspe) to reduce competitive state anxiety on karate athletes. J. Sportif 8, 169–188. doi: 10.29407/js_unpgri.v8i2.17807

[ref30] Hays K. Thomas O. Maynard I. Bawden M. (2009). The role of confidence in world-class sport performance. J. Sports Sci. 27, 1185–1199. doi: 10.1080/02640410903089798, PMID: 19724964

[ref31] Heatherton T. F. Wyland C. L. (2003). “Assessing self-esteem” in Positive psychological assessment: A handbook of models and measures. eds. Lopez S. J. Snyder C. R. (Washington, DC: American Psychological Association).

[ref32] Hill D. M. Shaw G. (2013). A qualitative examination of choking under pressure in team sport. Psychol. Sport Exerc. 14, 103–110. doi: 10.1016/j.psychsport.2012.07.008

[ref33] Holland M. J. Woodcock C. Cumming J. Duda J. L. (2010). Mental qualities and employed mental techniques of young elite team sport athletes. J. Clin. Sport Psychol. 4, 19–38. doi: 10.1123/jcsp.4.1.19

[ref34] Ishii H. Yanagiya T. Naito H. Katamoto S. Maruyama T. (2012). Theoretical study of factors affecting ball velocity in instep soccer kicking. J. Appl. Biomech. 28, 258–270. doi: 10.1123/jab.28.3.258, PMID: 21908898

[ref35] Jordet G. Hartman E. Visscher C. Lemmink K. A. (2007). Kicks from the penalty mark in soccer: the roles of stress, skill, and fatigue for kick outcomes. J. Sports Sci. 25, 121–129. doi: 10.1080/02640410600624020, PMID: 17127587

[ref36] Josefsson T. Ivarsson A. Gustafsson H. Stenling A. Lindwall M. Tornberg R.. (2019). Effects of mindfulness-acceptance-commitment (mac) on sport-specific dispositional mindfulness, emotion regulation, and self-rated athletic performance in a multiple-sport population: an RCT study. Mindfulness 10, 1518–1529. doi: 10.1007/s12671-019-01098-7

[ref37] Junge A. Dvorak J. Rosch D. Graf-Baumann T. Chomiak J. Peterson L. (2000). Psychological and sport-specific characteristics of football players. Am. J. Sports Med. 28, 22–28. doi: 10.1177/28.suppl_5.s-22, PMID: 11032104

[ref38] Junli L. Tianyuan L. Jinglan S. (2021). The impact of self-confidence, self-motivation and competitive state anxiety on attentional control in athletes in China. Rev. Psicol. Deporte 30, 31–48.

[ref39] Kimberlin C. L. Winterstein A. G. (2008). Validity and reliability of measurement instruments used in research. Am. J. Health Syst. Pharm. 65, 2276–2284. doi: 10.2146/ajhp070364, PMID: 19020196

[ref40] Krane V. (1994). The mental readiness form as a measure of competitive state anxiety. Sport Psychol. 8, 189–202. doi: 10.1123/tsp.8.2.189

[ref41] Li L. Jing L. Liu Y. Tang Y. Wang H. Yang J. (2023). Association of mindfulness with perfectionism, exercise self-efficacy, and competitive state anxiety in injured athletes returning to sports. Healthcare 11:2703. doi: 10.3390/healthcare11202703 37893777 PMC10606558

[ref42] Lochbaum M. Sherburn M. Sisneros C. Cooper S. Lane A. M. Terry P. C. (2022). Revisiting the self-confidence and sport performance relationship: a systematic review with meta-analysis. Int. J. Environ. Res. Public Health 19:6381. doi: 10.3390/ijerph19116381, PMID: 35681963 PMC9180271

[ref43] Lopes J. E. Jacobs D. M. Travieso D. Araújo D. (2014). Predicting the lateral direction of deceptive and non-deceptive penalty kicks in football from the kinematics of the kicker. Hum. Mov. Sci. 36, 199–216. doi: 10.1016/j.humov.2014.04.004, PMID: 24846289

[ref44] Macdonald H. Z. Olsen A. (2020). The role of attentional control in the relationship between mindfulness and anxiety. Psychol. Rep. 123, 759–780. doi: 10.1177/0033294119835756, PMID: 30866719

[ref45] Marchand W. R. (2012). Mindfulness-based stress reduction, mindfulness-based cognitive therapy, and Zen meditation for depression, anxiety, pain, and psychological distress. J. Psychiatr. Pract. 18, 233–252. doi: 10.1097/01.pra.0000416014.53215.86, PMID: 22805898

[ref46] Martinent G. Ferrand C. Guillet E. Gautheur S. (2010). Validation of the French version of the competitive state anxiety Inventory-2 revised (CSAI-2R) including frequency and direction scales. Psychol. Sport Exerc. 11, 51–57. doi: 10.1016/j.psychsport.2009.05.001

[ref47] Meland A. Ishimatsu K. Pensgaard A. M. Wagstaff A. Fonne V. Garde A. H.. (2015). Impact of mindfulness training on physiological measures of stress and objective measures of attention control in a military helicopter unit. Int. J. Aviat. Psychol. 25, 191–208. doi: 10.1080/10508414.2015.1162639, PMID: 27226703 PMC4867781

[ref48] Naderi A. Shaabani F. Zandi H. G. Calmeiro L. Brewer B. W. (2020). The effects of a mindfulness-based program on the incidence of injuries in young male soccer players. J. Sport Exerc. Psychol. 42, 161–171. doi: 10.1123/jsep.2019-0003, PMID: 32150722

[ref49] Noël B. Furley P. Van Der Kamp J. Dicks M. Memmert D. (2015). The development of a method for identifying penalty kick strategies in association football. J. Sports Sci. 33, 1–10. doi: 10.1080/02640414.2014.926383 24914924

[ref50] Norouzi E. Gerber M. Masrour F. F. Vaezmosavi M. Puehse U. Brand S. (2020). Implementation of a mindfulness-based stress reduction (Mbsr) program to reduce stress, anxiety, and depression and to improve psychological well-being among retired Iranian football players. Psychol. Sport Exerc. 47:101636. doi: 10.1016/j.psychsport.2019.101636

[ref51] Oguntuase S. B. Sun Y. (2022). Effects of mindfulness training on resilience, self-confidence and emotion regulation of elite football players: the mediating role of locus of control. J. Sport Exerc. Psychol. 2, 198–205. doi: 10.1016/j.ajsep.2022.08.003

[ref52] Orzech K. M. Shapiro S. L. Brown K. W. Mckay M. (2009). Intensive mindfulness training-related changes in cognitive and emotional experience. J. Posit. Psychol. 4, 212–222. doi: 10.1080/17439760902819394

[ref53] Serences J. T. Shomstein S. Leber A. B. Golay X. Egeth H. E. Yantis S. (2005). Coordination of voluntary and stimulus-driven attentional control in human cortex. Psychol. Sci. 16, 114–122. doi: 10.1111/j.0956-7976.2005.00791.x, PMID: 15686577

[ref54] Shepherd M. Findlay J. M. Hockey R. J. (1986). The relationship between eye movements and spatial attention. Q. J. Exp. Psychol. 38, 475–491. 10.1080/14640748608401609 3763952

[ref55] Short S. E. Short M. W. (2005). Differences between high-and low-confident football players on imagery functions: a consideration of the athletes’ perceptions. J. Appl. Sport Psychol. 17, 197–208. doi: 10.1080/10413200591010049

[ref56] Shrauger J. S. Schohn M. (1995). Self-confidence in college students: conceptualization, measurement, and behavioral implications. Assessment 2, 255–278. doi: 10.1177/1073191195002003006, PMID: 31368719

[ref57] Tang Y. Jing L. Liu Y. Wang H. (2023). Association of mindfulness on state-trait anxiety in choking-susceptible athletes: mediating roles of resilience and perceived stress. Front. Psychol. 14:1232929. doi: 10.3389/fpsyg.2023.1232929 37711325 PMC10497761

[ref58] Tang Y. Liu Y. Jing L. Wang H. Yang J. (2022). Mindfulness and regulatory emotional self-efficacy of injured athletes returning to sports: the mediating role of competitive state anxiety and athlete burnout. Int. J. Environ. Res. Public Health 19:11702. doi: 10.3390/ijerph191811702, PMID: 36141969 PMC9517234

[ref59] Thienot E. Jackson B. Dimmock J. Grove J. R. Bernier M. Fournier J. F. (2014). Development and preliminary validation of the mindfulness inventory for sport. Psychol. Sport Exerc. 15, 72–80. doi: 10.1016/j.psychsport.2013.10.003, PMID: 32757693

[ref60] Tomé-Lourido D. Arce C. Ponte Fernández D. (2019). The relationship between competitive state anxiety, self-confidence and attentional control in athletes. Rev. de Psicol. del Deporte 28, 143–150.

[ref61] Tuğlu Ş. Ermiş E. Erilli N. A. (2022). Evaluation of pressure elements in the penalty kicks. Pakistan J. Medical Health Sci. 16:901. doi: 10.53350/pjmhs22165901

[ref62] Van Der Kamp J. (2006). A field simulation study of the effectiveness of penalty kick strategies in soccer: late alterations of kick direction increase errors and reduce accuracy. J. Sports Sci. 24, 467–477. doi: 10.1080/02640410500190841, PMID: 16608761

[ref63] Vella-Fondacaro D. Romano-Smith S. (2023). The impact of a psychological skills training and mindfulness-based intervention on the mental toughness, competitive anxiety, and coping skills of futsal players—a longitudinal convergent mixed-methods design. Sports 11:162. doi: 10.3390/sports11090162, PMID: 37755839 PMC10536553

[ref64] Walker S. (2019). Negative self-appraisal mediates the relationship between mindfulness and confidence among adolescent female provincial hockey players in South Africa. S. Afr. J. Sports Med. 31:4371. doi: 10.17159/2078-516X/2019/v31i1a4371, PMID: 36817994 PMC9924597

[ref65] Wilson M. R. Wood G. Vine S. J. (2009). Anxiety, attentional control, and performance impairment in penalty kicks. J. Sport Exerc. Psychol. 31, 761–775. doi: 10.1123/jsep.31.6.761, PMID: 20384011

[ref66] Wood G. Jordet G. Wilson M. R. (2015). On winning the “lottery”: psychological preparation for football penalty shoot-outs. J. Sports Sci. 33, 1758–1765. doi: 10.1080/02640414.2015.1012103, PMID: 25687286

[ref67] Wood G. Wilson M. R. (2010). A moving goalkeeper distracts penalty takers and impairs shooting accuracy. J. Sports Sci. 28, 937–946. doi: 10.1080/02640414.2010.495995, PMID: 20568032

[ref68] Yantis S. (1993). Stimulus-driven attentional capture and attentional control settings. J. Exp. Psychol. Hum. Percept. Perfom 19, 676–681. doi: 10.1037/0096-1523.19.3.676 8331320

[ref69] Young W. R. Ellmers T. J. (2022). “Translating attentional control theory to applied psychological eye tracking research” in Eye tracking: Background, methods, and applications. ed. Stuart S. (Springer US: New York, NY).

[ref70] Zadkhosh S. M. Zandi H. G. Hemayattalab R. (2018). Neurofeedback versus mindfulness on young football players anxiety and performance. Turk. J. Kinesiol. 4, 132–141. doi: 10.31459/turkjkin.467470

